# Impact of a “Digital Health” Curriculum on Students’ Perception About Competence and Relevance of Digital Health Topics for Future Professional Challenges: Prospective Pilot Study

**DOI:** 10.2196/58940

**Published:** 2025-05-12

**Authors:** Juliane Kröplin, Leonie Maier, Jan-Hendrik Lenz, Bernd Romeike

**Affiliations:** 1 Department of Oral and Maxillofacial Surgery, University Medical Centre Rostock, Schillingallee 35, Rostock, 19057, Germany, 49 3814946649; 2 Department of the Dean of Studies in Medical Didactics, University Medical Centre Rostock, Rostock, Germany

**Keywords:** education, gamification, robotics, telemedicine, wearable electronic devices, artificial intelligence

## Abstract

**Background:**

The rapid integration of digital technologies in health care has emphasized the need to ensure that medical students are well-equipped with the knowledge and competencies related to digital health.

**Objective:**

This study aimed to evaluate the impact of the “Digital Health” curriculum at our university on the perceptions of medical students regarding the relevance of digital health topics for their future professions and their self-assessed competence in these areas.

**Methods:**

The “Digital Health” curriculum was introduced at a German university for 2 consecutive semesters. The perceived relevance of topics for their future careers and their subjective competence were evaluated before and after the curriculum using a Likert scale. Furthermore, the practical gamification-based teaching part of the robotics teaching unit was evaluated. In total, 6 months after completing the last semester, a follow-up analysis was performed with questions on the significance of the completed curriculum for current and future professional challenges regarding digital health and suggestions for improvement for innovative teaching. The study was meticulously planned and supported by an approved ethics vote of the local ethics committee to ensure that all ethical guidelines were adhered to (A 2022-0137).

**Results:**

A total of 20 students participated, with 13 (65%) being women. In particular, data protection and information security were considered the most relevant topics both before and after the curriculum. Significant increases in perceived importance were observed for messenger apps (mean increase of 0.8 [SD 1.2]; *P<*.01). Regarding self-assessed competence, significant development was observed on almost all topics. The greatest development was observed in robotics (mean increase of 1.8 [SD 1.2]; *P*<.001), open educational resources (mean increase of 1.7 [SD 1.5]; *P*<.001), and simulation-training (mean increase of 1.6 [SD 1.3]; *P*<.001). The gamification-based, robot-related teaching was predominantly rated suitable and very enjoyable for the students.

**Conclusions:**

The results highlight the potential to integrate more innovative teaching techniques, such as gamification, augmented reality, virtual reality, and simulation training, into a technologically advanced health care environment. Finally, the overarching importance of artificial intelligence and digital health applications signals the need to further integrate them, given their potential in remote and personalized medicine.

## Introduction

The term “digital health” closely links advanced medical technologies, disruptive innovations, and digital communication with the provision of optimal health care [1]. Providing medical students with a comprehensive understanding of the basic concepts and possibilities of eHealth is crucial to enabling future physicians to recognize and apply the benefits of these services in their daily practice. It is likely to increase their openness to digital health and reduce their reluctance to adopt digital health [2].

Medical students are well aware of the possible applications and effects of digitization, including artificial intelligence (AI) in medicine [3]. Despite their positive attitude and high expectations, they express concerns about patient acceptance and possible professional consequences [4]. Through an effective curriculum, students can gain learning benefits from this technology and understand both opportunities and challenges [5]. Most medical students consider this training to be a valuable part of their education, as it helps them better understand and apply telemedicine approaches with greater confidence [6].

There is a general consensus in the current literature that future health care professionals must be prepared for the digital age [7]. Elective subjects appear to be suitable formats for prompt introduction, but long-term implementation in the compulsory curriculum should be the goal [8]. National initiatives could better meet the challenges of digitization in health care and offer solutions on how best to teach digital skills to students [9]. Innovative teaching content also requires innovative teaching methods. A promising approach is gamification, which refers to the integration of playful elements in a serious context. Educators in the health care sector are also increasingly relying on gamification to optimize students’ learning success [10].

Current developments raise fundamental questions about the future of health care professions: (1) What digital skills will be needed in the future? (2) How will digitization affect the relationship between patients and health care professionals ? And (3) adapting education and training in health care professions will be crucial to meet the demands of an increasingly digitalized society [11].

The curriculum “Digital Health - Digitization and Digital Transformation of Medicine” was offered for the first time at a German university in the winter semester of 2022 or 2023. An analysis of the implementation process was published recently [12].

The curriculum is divided into four main topics: (1) digital transformation of medical education, (2) digital communication, (3) digital leadership, and Robotics or AI.

The study aims: (1) to analyze the impact of a transdisciplinary digital health curriculum on students’ perceived relevance and competence regarding digital health topics and (2) to analyze the acceptance and suitability of a gamification-based teaching method integrated into the existing curriculum.

This was intended to evaluate when and how the opinions and subjective experiences of medical students can be optimally incorporated and integrated into the development of an effective curriculum. By integrating students into this process, the aim is to ensure that their individual perspectives are considered and a user-orientated curriculum is created.

## Methods

### Study Structure and Design

This prospective pilot study was designed as a pre-post analysis to assess the development in subjective competence and the subjectively perceived impact of our digital health curriculum on the perception about the relevance of digital health topics for future professional challenges. The curriculum content and implementation process have already been published [12].

Participation in both the curriculum and all evaluations was voluntary. The data were collected and analyzed anonymously.

### Participants

The age and gender of the curriculum participants were analyzed. Students were also asked whether they were interested in doing a doctorate and what plans they had for a postdoc phase.

### Relevance and Competence Acquisition Concerning Digital Health Topics: Pre-Post Comparison

The digital health curriculum included the following topics: big data, AI, digital health apps, messenger apps, wearables, telemedicine, data protection or information security, digital ethics, simulation training, augmented reality (AR), virtual reality (VR), economy, e-learning, robotics, digital leadership, mobile working, social media, and open educational resources (OER.

In the first step, students were asked to rate the relevance of individual course content for their future career on a Likert scale from 1 (very relevant) to 5 (irrelevant; Table 1). The subjective assessment of their own competence was also carried out on a Likert scale from 1 (very good) to 5 (not good at all) on the same topics (Table 2). The same survey was carried out again at the end of the course to achieve the aim of a pre-post comparison.

**Table 1.** Questionnaire of gamification-based robotic teaching.

Question	Answer options
RT^a^1: How did you complete the learning unit with the robot construction kit?	Alone In teams of two With other teams of two of the digital health curriculum With other people (parents, friends, etc)
RT2: How much time in hours did you spend with the robot construction kit?	Building Programming
RT3: What applications have you carried out?	Building the robot Yes No Programming movements Yes No Programming of acoustic signals Yes No Programming of visual signals Yes No
RT4: By using the robot construction kit, I programmed independently for the first time	Yes No
RT5: The course was a suitable way to reduce reservations about the topic of robotics in medicine	Very true Rather true Partly/partly Rather not applicable Does not apply at all
RT6: I enjoyed working with the robot construction kit	Very true Rather true Partly or partly Rather not applicable Does not apply at all

a RT: robotic teaching.

**Table 2.** Follow-up evaluation 6 months after the end of the course.

Question	Answer option
Follow-up question 1: Define the term “digital health.”	—^a^
Follow-up question 2: How do you think your future work will be changed by developments in digital health?	—
Follow-up question 3: Do you see these changes in a positive or negative way?	Positive Negative Neither (neutral)
Follow-up question 4: Give 2 examples each of positive and negative changes	Positive: Negative:
Follow-up question 5: How do you think teaching should be adapted?	—
Follow-up question 6: For example, have you found the elective’s Instagram account useful and did you use it?	Useful and used it Useful, but didn’t use it Not useful, didn’t use it I did not know about the account
Follow-up question 7: Do you already use parts of Digital Health (both in general and for learning)? eg, wearables for interpreting your own medical data, AR^b^ and VR^c^ in the medical field, ChatGPT, LLM^d^ (other generative AI^e^)	Yes (please describe): No

a not applicable.

b AR: augmented reality.

c VR: virtual reality.

d LLM: large language model.

e AI: artificial intelligence.

### Gamification in Robotic Teaching

In order to introduce the students to the subject of robotics playfully and reduce their fear of contact, they were provided with a robot construction kit for the entire duration of the course (Lego Mindstorms, Billund, and Denmark). Building instructions can be found online [13].

**Figure 1.**
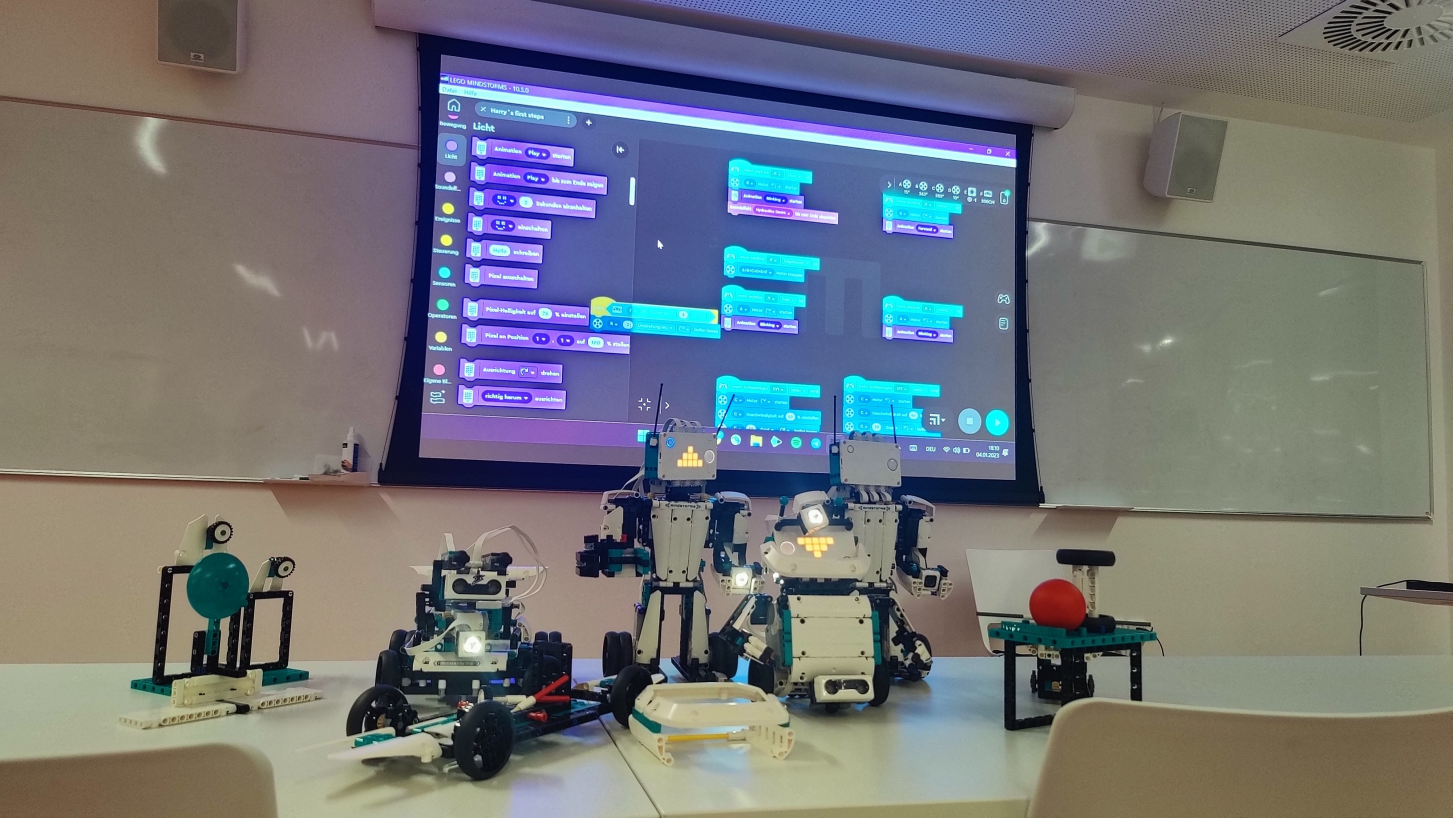
Different robots built with the Lego Mindstorms construction kit.

**Figure 2.**
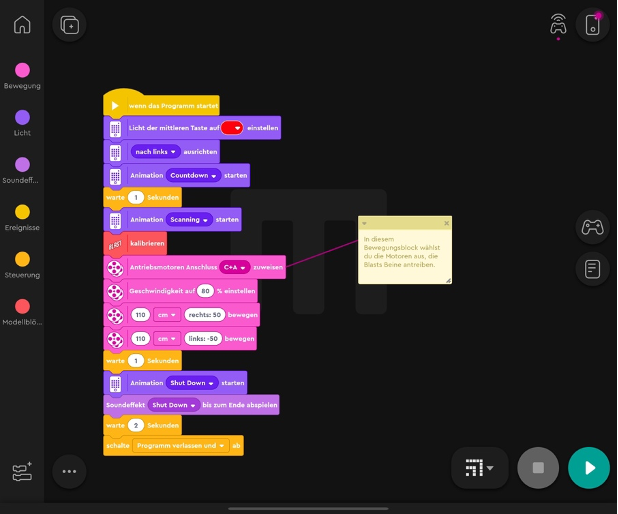
Screenshot of the Lego Mindstorms application-based visual programming software.

They were able to build a robot at home. During one course, the robots were demonstrated and reprogrammed live. Figure 1 shows 4 robots, that were built by the students during the semester. Students could program movements, and acoustic and visual signals with an application-based visual programming software, that can be downloaded for free (Lego Mindstorms app, Billund, and Denmark [14]). Due to the visual programming environment, users create programs by dragging and dropping adaptable command blocks into a sequence (Figure 2).

The gamification analysis took place at the beginning of the robotics teaching (RT) unit (course days 10 and 12). The Lego Mindstorms kit enabled students to built different types of robots. They had also the choice to build the robot alone or in teams. There were no specific instructions.

An intensive literature search found no validated, usable questionnaires, which is why our own questionnaires were developed as part of this pilot study.

The students’ experiences were assessed using a 6-item questionnaire with open and closed questions (Robotics R-questions Table 1).

The application behavior was analyzed and the usefulness of the teaching method was assessed.

### Follow-Up Evaluation

In total, 6 months (cohort 1) and 1 year (cohort 2) after completion of the curriculum, participants were asked to participate in a retrospective online survey using the online tool SurveyMonkey [15] (follow-up questions, Table 2).

This survey aimed to capture the sustainable subjective perception of various aspects related to the implementation of digital health in medical education and professional practice (Table 2). Participants were asked about their opinions on the future impact of digital health on their work and whether they felt positive or negative about these changes. Another focus was on the participants’ opinions on possible adaptations of medical teaching to the developments in digital health. Furthermore, usage practices in the field of digital health were recorded both in general and in the context of learning to gain insight into the current integration of these technologies.

### Statistics

Data were analyzed using IBM SPSS version 27. Quantitative data like pre-post-test results were analyzed with paired, 2-tailed Student’s *t* test. A value of *P*<.05 was considered statistically significant. Open questions like the participants’ career goals, definition of the term “digital health,” their opinion about how future work would be changed by developments in digital health, or how teaching should be adapted were summarized with descriptive statistics.

### Ethical Considerations

The study was meticulously planned and supported by an approved ethics vote of the Ethics Committee at the Medical Faculty of the University of Rostock to ensure that all ethical guidelines were adhered to (A 2022‐0137). Participation in the evaluation was voluntary. Informed consent to participate was obtained from all of the participants.

## Results

### Participants

Within the first year, a total of n=20 students participated in the digital health curriculum. Out of 20 students, 13 (65%) participants were women and 7 (35%) men. During the pre-evaluation, the participants’ academic career goals were evaluated. A total of 90% (18/20) of the participants aspire to a doctorate and 10% (2/20) of the participants also intend to obtain a PhD degree.

### Relevance and Competence Concerning Digital Health Topics: Pre-Post Comparison

Table 3 shows the results of the pre-post comparison on the subjectively assessed relevance of the content of the digital health elective for the participants’ later professional future. The topic of data protection and information security was rated the most important at the beginning of the curriculum (mean 1.2 [SD 0.5]). This assessment was also confirmed after the completion of the curriculum. A significant increase in importance was verified for the topic of messenger apps.

Table 4 shows the results of the pre-post comparison of the participants’ subjective assessment of their own competence regarding the learning objectives of the curriculum. Except for the topics of messenger apps and social media, there was a significant subjective increase in knowledge in all topics. The greatest increase in competence was in the area of robotics; the least in the area of messenger apps. The topics in which no significant increase in knowledge could be generated were also those with the highest score in previous knowledge during the initial evaluation.

**Table 3.** Assessment of the relevance of curriculum content for the later professional future.

Relevance for the future	Pre,mean (SD)	Post,mean (SD)	Relevance increase, mean (SD)	*P* value
Messenger apps	2.9 (1.1)	2.1 (1.0)	0.8 (1.2)	.01
AR^a^ and VR^b^	2.1 (1.0)	1.8 (1.2)	0.3 (1.3)	.33
Mobile working	2.4 (1.2)	2.1 (1.3)	0.3 (1.6)	.40
Wearables	2.1 (1.2)	2.4 (1.1)	0.3 (1.6)	.37
Digital health apps	1.8 (0.9)	1.6 (1.0)	0.2 (1.5)	.55
OER^c^	2.0 (1.0)	1.8 (1.0)	0.2 (1.5)	.57
Big data	2.1 (0.9)	2.0 (1.1)	0.1 (1.0)	.65
Economy	1.9 (1.0)	1.8 (1.0)	0.1 (1.9)	.58
AI^d^	1.5 (0.7)	1.5 (1.0)	0 (1.1)	.84
Social media	2.5 (1.0)	2.5 (1.4)	0 (1.3)	.87
Data protection orinformation security	1.2 (0.5)	1.3 (0.8)	−0.1 (1.0)	.65
Digital didactics	1.7 (1.0)	1.8 (1.2)	−0.1 (1.2)	.58
Digital ethics	1.4 (0.8)	1.5 (0.9)	−0.1 (0.9)	.61
Digital leadership	1.6 (0.7)	1.7 (1.0)	−0.1 (1.1)	.55
Simulation training	1.4 (0.5)	1.5 (1.0)	−0.1 (1.0)	.53
Robotics	1.5 (0.8)	1.8 (1.1)	−0.3 (1.0)	.13
Telemedicine	1.8 (1.1)	2.1 (1.4)	−0.3 (1.6)	.51

a AR: augmented reality.

b VR: virtual reality.

c OER: open educational resources.

d AI: artificial intelligence.

**Table 4.** Subjective competence development determined via a pre-post-comparison.

Competence	Precomparision, mean (SD)	Postcomparision, mean (SD)	Knowledge increase, mean (SD)	*P* value
Robotics	4.2 (1.1)	2.4 (1.0)	1.8 (1.2)	<.001
OER^a^	3.7 (1.3)	2.0 (0.8)	1.7 (1.5)	<.001
Simulation training	4.1 (1.1)	2.5 (1.2)	1.6 (1.3)	<.001
AI^b^	3.8 (1.1)	2.3 (0.9)	1.5 (1.0)	<.001
Digital leadership	4.2 (1.1)	2.7 (1.0)	1.5 (1.2)	<.001
AR^c^ and VR^d^	3.6 (1.4)	2.2 (1.1)	1.4 (1.6)	<.001
Mobile working	3.9 (1.2)	2.5 (0.9)	1.4 (1.2)	<.001
Wearables	3.6 (1.2)	2.2 (1.2)	1.4 (1.9)	.004
Digital health apps	3.2 (1.4)	1.9 (0.9)	1.3 (1.4)	<.001
Digital didactics	3.0 (1.2)	1.8 (1.0)	1.2 (1.4)	.00
Digital ethics	3.8 (1.0)	2.7 (1.0)	1.1 (1.1)	<.001
Telemedicine	3.9 (1.0)	2.8 (1.2)	1.1 (1.3)	<.001
Data protection orinformation security	3.3 (1.3)	2.4 (1.1)	0.9 (1.3)	.01
Big data	4 (1.4)	3.2 (0.9)	0.8 (1.5)	.02
Economy	3.8 (1.2)	3.0 (1.2)	0.8 (1.5)	.02
Social media	2.1 (1.2)	1.9 (1.1)	0.2 (1.4)	.54
Messenger apps	1.8 (1.2)	1.6 (1.1)	0.2	.56

a OER: open educational resources.

b AI: artificial intelligence.

c AR: augmented reality.

d VR: virtual reality.

### Gamification in Robotic Teaching

A total of 13 students participated in the robotics evaluation of the practical teaching unit. All students worked on the task in teams (RT1). The average time that participants invested in the task was 2 hours and 30 minutes (1.48 h building the robot; 0.42 h programming the robot; RT2). In Figure 1, 11 students had built their robot at the time of the survey; n=7 had already programmed the robot (RT3). For the vast majority of students (n=11), this was their first opportunity to generate code independently (RT4). Most of the participants (n=10) rated the application of the robotics teaching unit as suitable (RT5) and enjoyed using it (RT6).

### Follow-Up Evaluation

In total, 13 out of 20 (65 %) students from both semesters of the curriculum participated in our follow-up evaluation. The first question of the participants, follow-up question 1, confirmed a basic understanding of the former participants’ definition of digital health. The most frequently mentioned key points were digital transformation or digitization of medicine and the improvement of health care through digital technologies. Table 5 shows all responses to follow-up question 2 of how digital health will influence future work. The most common expectation is about new possibilities in diagnostics and therapy.

All survey participants are positive about the changes associated with digital health (follow-up question 3). The most frequently cited potential positive change was an increase in efficiency or increased flexibility and new possibilities in diagnostics and therapy (n=7 each; follow-up question 4). The most frequently cited potential negative change was an increased risk of misusing and stealing data (n=8) and the risk of digital addiction (n=5; follow-up question 4).

In follow-up question 5, former participants were asked to provide their assessment of the extent to which teaching should be adapted to the challenges of digital transformation. In particular an adaptation of the learning methodology (n=6); especially AI-supported, for more individualized or efficient or sustainable learning), familiarization with existing apps (n=5); especially digital documentation systems) and the general inclusion of digital health subjects (n=4) was indicated.

At the beginning of the curriculum, all participants were informed of the existence of a social media channel. As part of the follow-up evaluation, the students were able to evaluate both their usage behavior and the usefulness of this channel (follow-up question 6; Table 6).

The follow-up question (7 about the current use of digital tools in students’ everyday medical lives was answered by all survey participants. When asked about specific applications, n=12 participants confirmed the use of ChatGPT [16] and the translation software DeepL [17]. Other participants indicated the use of wearables (n=5) and AR or VR for learning anatomy (n=1).

**Table 5.** Follow-up question 2: How do you think your future work will be changed by developments in digital health? Core messages; n=x.

Answer to follow-up question 2	Quantity (n)
New possibilities in diagnostics and therapy (eg, AI-assisted)	8
Easier data processing and documentation (eg, digital patient file)	4
Acceleration of processes or more efficient work	4
Home office or flexibility (eg, video consultation)	3
Generally positive enrichment (better patient contact)	2
Increased risk of data misuse and theft	1

**Table 6.** Follow-up question 6: Have you found the elective’s Instagram account useful and did you use it, n=x?

Answer to follow-up question 6	Quantity (n)
Useful and used it	3
Useful but did not use it	8
Not useful, did not use it	1
I did not know about the account	1

## Discussion

### Principal Findings

This study provides an overview of the influence of our digital health curriculum on students’ perception about their competence and the relevance of digital health topics for their future professional challenges. The results are based on 2 consecutive semesters in which the content of the elective subject “Digital Health” was taught at a German university. Although the topic of data protection was named the most relevant content, the students confirmed a significant increase in relevance for numerous topics during the curriculum, primarily with regard to the teaching unit messenger apps.

The analysis of the practical teaching unit (building and programming a LEGO robot) has shown that this approach is fun and was rated by the students as suitable for reducing fear of contact with the topic of robotics.

In a follow-up online analysis 6 months after completing the course, the students stated, among other things, that they would like to see a general adaptation of the teaching methods in the degree program, for example, to deepen individual, more efficient, and sustainable learning through AI-based apps.

### Demographics

A particularity of our cohort appears to be a very high interest in science and in completing a doctoral thesis, while globally, medical students rather underscore the necessity and importance of research in medical education, with many students reporting positive attitudes and interest in research efforts [18].

### Relevance and Competence Acquisition Concerning Digital Health Topics: Pre-Post Comparison and Follow-Up Evaluation

#### Data- and IT-Security

Data protection and IT security were the highest ranking in terms of relevance among our students. Students also reported a significant increase in competence after completing the curriculum.

As part of the follow-up analysis, the students rated data protection and information security as the most frequently cited potentially negative change. In particular, data misuse and data theft were identified as risk factors. This can be explained, among other things, by the presence of the subject and the actual threat scenarios [19]. Furthermore, the current literature shows the need to protect health care technology and patient data against unauthorized access [20]. In the context of relevance analyses on the topic of digital health, the topic of data protection generally has the highest priority [21]. The constant assessment of high relevance in the pre- and post-test survey indicates that we have adequately addressed the topic in the curriculum.

#### Evolving Telehealth Landscape

Although telemedicine has existed in various forms for decades, recent technological innovations, combined with changes in patient preferences and the global response to crises such as the COVID-19 pandemic, have greatly accelerated its adoption and importance [22]. Data from our curriculum suggest that, while students recognize the importance of many aspects of digital health, there seems to be an underestimate of the growing importance of telehealth. Telemedicine not only expands access to health care but also introduces efficiencies that traditional in-person visits may lack. Current and future health care professionals need a solid understanding of these tools, both for their practical utility and for their wider impact on the health care system. The practical application of the interaction of telemedicine is an aspired goal for the further development of our curriculum. Simulated real-life experience would allow us to directly experience both the advantages and possible hurdles of communication.

#### Ethical Aspects of Digital Health

As our results show, the ethics subject area was rated as very important by the participants in the curriculum. Furthermore, the students confirmed a significant subjective increase in competence as a result of their participation. Ethical considerations of digital health are manifold. Issues such as consent in the age of digital monitoring, bias in AI algorithms, and ethical use of patient data for research are all pertinent discussions that must be ingrained in the medical curriculum. Furthermore, ethical perspectives shall not be limited to the technology itself. Instead, it is suggested to consider the broader sociotechnical system and also social justice for a comprehensive ethical analysis of digital health [23]. It can be assumed that the increasing use of AI in medical practice will also change the way future physicians understand their role. This also affects the development of as yet unforeseeable areas of responsibility, which require adaptation of medical training. Therefore, it is particularly important to prepare students for these changes in order to ensure high-quality health care [24].

#### Modern Pedagogies: AR VR, Simulation Training and OER

The digital health curriculum saw a significant increase in the perceived competence of students in areas such as simulation training. Simulation, particularly in medical education, offers a risk-free environment for students to hone their skills, make mistakes, and learn in a controlled setting . Furthermore, the rise of OER democratizes learning and offers students a plethora of resources that were not available to previous generations [25]. During the analysis period, we succeeded in significantly increasing the subjective competence gained on the topic of OER.

Taking into account the study results of Hassall et al [25], it can be assumed that students will also make use of OER resources in the future. In this study, a lack of awareness, motivation, and training regarding OERs were identified as the biggest obstacles to the use of OERs.

The students also confirmed a subjective increase in knowledge with regard to the topic of VR and AR. VR and AR technologies have particular potential with regard to modern pedagogies [26]. They address the need for sustainable medicine and enable a reproducible and objectifiable learning environment.

Taking the follow-up analysis into account, we feel that we will offer the compulsory elective subject in the future. However, the focus will be on further adaptation of teaching methods (digital transformation of medical education) as well as the increased teaching of existing apps for digital documentation systems.

#### Digital Health in Social Media

Social media has become an increasingly important source of information on medical content in recent years. This also applies to teaching [27]. This has prompted us to accompany our digital health curriculum with a social media channel. The results related to our digital health curriculum Instagram account are of particular interest to us. The high confirmation of the usefulness of such a digital communication tool is remarkable because the use of social media in medical education is an emerging field that needs further investigation and is still the subject of controversial debates [28,29]. The fact that only a minority used the channel can be explained by the fact that it was preferably used for general information on special offers on the compulsory elective subject. In the future, expanding the interaction with participants and the possibility of active participation to solve specific tasks would be a way to increase the frequency of use.

#### AI

AI plays an increasing role in medical education [30]. The great importance that students continue to attach to AI for their professional future in a pre-post comparison shows the special significance of this topic. This makes it even more significant that the students experienced a significant subjective increase in competence as a result of participating in the digital health curriculum. The follow-up analysis also provides an impressive insight into the current usage behavior of students with regard to AI. In particular, the use of ChatGPT and the translation software DeepL was mentioned. A development that is in agreement with the currently available literature and is of great interest for the further development of future teaching and research [31,32]. Furthermore, the follow-up analysis shows the students’ desire for adapted teaching using AI-based support systems. These findings once again demonstrate the particular importance of analyzing the necessary framework conditions for constructive ethical use of AI.

#### Gamification in Robotic Teaching

It can be assumed that the topic of robotics was evaluated with the greatest subjective increase in competence due to the special focus on the implementation of a gamification-based teaching set.

Robot-assisted surgery is one of the most revolutionary developments in surgery. It can be assumed that demand will continue to increase in the future. Recently, the performance of robotic surgery has increased exponentially. This is primarily due to the benefits of robotics. Robotic surgery can help reduce hospitalization and recovery times. In addition, innovative robotic technology enables better visualization and fewer postoperative complications. Access to robotics for students is often limited by the fact that cost-intensive technology is not available at all locations [33]. Furthermore, robotics training is offered predominantly only to experienced surgeons and is mainly based primarily on workshops and fellowships [34]. Stockheim et al [34] have already addressed residents as a target group with their RoC curriculum, Robotic Curriculum for Young Surgeons. Introducing students at an early stage has the potential to reduce fears of contact even before they start their specialist training and to get students excited about specialisms with robotic apps.

Considering the limited access for students, it was particularly important to develop solutions that enable low-threshold access to the topic of robotics. The success of this decision is reflected largely in the results of our evaluation.

Most of our participants considered the robot construction kit to be a suitable way of reducing the fear of contact with the subject of robotics. The majority of students also stated that working with the robot was fun. These results underline the findings of previously published literature on the subject of gamification [35].

In medical education, in particular, gamification has been shown to lead to a higher level of motivation, commitment, and challenge compared to traditional didactics [36]. This offers teachers alternative ways to optimize the teaching process. This way of imparting skills is widely received by learners and allows for an immersive experience that is perceived as effective, engaging, easy to understand, interesting, and instructive [37]. Research even suggests that gamification can improve the knowledge of health care professionals compared to traditional training [ 38].

### Limitations

Limitations arise in particular from the single-center study design and the small number of participants at the time of analysis. However, the number of participants is quite normal for a pilot study and a compulsory elective, where students work in small groups in order to increase the quality of the lessons. Finally, it must be considered that the results are purely subjective assessments of the participants. A follow-up study will also ask about the acquisition of specialist knowledge.

### Conclusions

The introduction of the “Digital Health: Digitization and Digital Transformation of Medicine” curriculum stands as proof of the changing face of medical education in the 21st century. Positive outcomes in terms of competence development and alignment with relevant digital health topics underscore its success. Although the curriculum effectively improved competencies on a range of topics, emphasis on data security and ethics is paramount given the vulnerabilities associated with digital health data.

The integration of gamification-based teaching methods can be seen as a useful addition to the teaching of basic skills and a low-threshold approach to complex digital topics.

As digital health continues to advance, the task is now to ensure that such curricula are not static but evolve in tandem with the technological and ethical landscape of modern healthcare.

## Abbreviations

AI: artificial intelligence
AR: augmented reality
OER: open educational resources
RT: robotic teaching
VR: virtual reality
